# Magneto-optical Kerr effect in surface engineered 2D hexagonal boron nitride

**DOI:** 10.1038/s41598-022-14594-z

**Published:** 2022-06-28

**Authors:** Ziba Torkashvand, Kavoos Mirabbaszadeh, Farzaneh Shayeganfar, Changgu Lee

**Affiliations:** 1 Department of Physics and Energy Engineering, Amirkabir University of Technology, Tehran, 15875-4413 Iran; 2 School of Mechanical Engineering, Sungkyunkwan University, Suwon, 16419 South Korea

**Keywords:** Energy science and technology, Materials science, Nanoscience and technology, Optics and photonics, Physics

## Abstract

Magnetism in atomically thin functional materials can be an important phenomenon for exploring two-dimensional magneto-optics. Magneto-optical experimental data have revealed significant Kerr signals in insulator thin films. Here, the magneto-optical Kerr effect of oxygen functionalized and doped hexagonal boron nitride (hBN) has been investigated by performing first-principles calculations. We calculated Kerr angle and Kerr ellipticity for functionalized hBN as an attention-drawn material. Moreover, increasing of oxygen doping percentage leads to the introduction of surface plasmon to hBN. Our findings show that the functionalized hBN can tolerate high-temperature conditions, keeping oxygen atoms bridge-bonded. These giant opto/magnetic responses of insulating 2D materials provide a platform for the potential designing of magneto-optical devices.

## Introduction

The magneto-optical (MO) effect as the output of interaction of magnetic materials with light has been characterized experimentally by measuring polarization state, phase, and intensity of light^1^. Magneto-optical Kerr effect (MOKE), rotation of polarization plane of reflected light from magnetic substrates, has been used as a means of observing magnetic and electronic properties^1^. A recent discovery of two-dimensional (2D) magnets by using MOKE revealed their magneto-optical properties^2,3^ and aroused interest in applications in sensors, filters, and polarizers to nonlinear optical devices^4^.

The atomically thin 2D materials with diverse opto-electronic properties demonstrate the interplay of large shape anisotropy based on featuring the thickness of a few nanometers as well as anisotropy in opto-magnetic properties, which provide unlimited possibilities for the MO applications^1^. Among 2D materials, hBN is the most stable boron nitride structure^5,6^, the best graphene counterpart, large band-gap material, and a promising structure for opto-electronic and nanophotonic applications^7–9^, without dangling bonds and charge traps^10,11^. Furthermore, hBN successively has been used as dielectric gate material^12,13^. Also, it has been shown that graphene devices on hBN dielectric possess enhanced stability, atomically smooth surface, intrinsic potential, and also lower scattering charge traps in comparison with graphene devices on SiO2 dielectric so it is an evidence for the most comprehensive dielectric layer used so far for graphene electronics^14,15^. The density of nanoparticles on the surface of boron nitride nanosheet (BNNS) can be controlled accurately through the synthesis adjustment and changing the reaction parameters^16^. Li et al.^17^ in a comprehensive DFT calculation, have considered the atomic structure and magnetic properties of configurations of a wide range of transition metals adsorbed on single-layer boron nitride. They divided these elements into two groups: one with adsorption energies lower than 0.5 eV and the other with higher ones which are following physical and chemical adsorption processes, respectively. Therefore, they concluded that this behavior is strongly related to the occupation of electrons in the electronic structure of each atom.

Searching for a reliable method for chemical functionalization without using poisoning chemicals and avoiding structural deformation leads to the plasma treatment due to its powerful ability for surface modification and adding active functional groups with the help of plasma^18,19^. Nevertheless, there is limited number of reports of plasma treatment on 2D materials but very few on hBN especially in the form of monolayer as a more sensitive structure to the energetic Ions. Singh et al.^20^ reported  100 fold improvement of electrical resistance based on optical and electrical measurements for $O_2$ plasma-treated few-layer hBN.

Also, it has been shown that hydrogenation can tune the electronic structure of hBN sheets by inducing extra interactions between N $2P_z$ and *H* 1S orbitals, which is beyond what occurs in the pristine hBN^21,22^. BNNS during chemical reactions with Au nanoparticles shows reduced band-gap and the linear relationship between conductance and Au concentration, which is the evidence of the Lewis base interactions of boron atoms and amine functionalized Au nanoparticles^16^. As Xiong et al.^23^ have found that an adsorption behavior directly depends on the electronic properties of hBN.

Investigating Magneto-optical related properties of materials has been extensively welcomed in recent years due to not only the necessity of high-density magnetic data storage^24^ but also the high potential of Kerr effect in materials research possessing high sensitivity and high accuracy without loss of the magnetism in low dimensional materials^1^.

2D magnetic materials are attracting attention in the field of magnetism and spintronics due to the potent ability of electrical control reinforcement on electromagnetic devices^25^. Huang et al. reported on magneto-optical Kerr effect (MOKE) microscopy of monolayer chromium triiodide $(CrI_3)$ for the first time as an indication of the ferromagnetic nature of the first magnetic 2D material^3^. DFT calculations are extensively used on MOKE for 2D materials and Heusler alloys, revealing the effect of strongly bounded excitons of charge-transfer on magneto-optical properties, interband transitions, spin-orbit coupling, and half-metallic geomagnetism in such magnetic materials^24,26–28^.

Here, we propose a computational framework enabling one to unveil magnetic properties of non-magnetic nature materials such as hBN in terms of doping and surface engineering, comparing its magnitude with the intrinsic magnetic materials. We have extensively investigated the electronic and optical properties of these materials. Furthermore, For the first time we report on the calculation of MOKE for the surface engineered hBN nanostructures as nonmagnetic and extrinsic ferromagnetic 2D materials using DFT calculations to assert that magneto-optical Kerr rotation is a reliable technique to evaluate the magnetic properties of materials as it has been used so far for the determination of the level of ferromagnetism in new-found materials theoretically and experimentally (Table 1).

**Table 1:** Relaxed structural parameters for pristine hexagonal boron nitride and four different percentage of oxygen functionalization.

Structure	a = b (Å)	$\gamma \,(^{\circ })$	Buckling (Å)	$E_b$ (eV)	$E_g$ (eV)
Pristine	5.027	120	0	–	4.670
$\theta$ = 1/8	5.077	120.05	0.28	− 3.74	3.213
$\theta$ = 2/8	5.172	119.94	0.29	− 5.26	2.432
$\theta$ = 3/8	5.304	121.08	0.29	− 7.39	2.616
$\theta$ = 4/8	5.439	121.59	0.20	− 9.83	2.792

## Results

**Figure 1:**
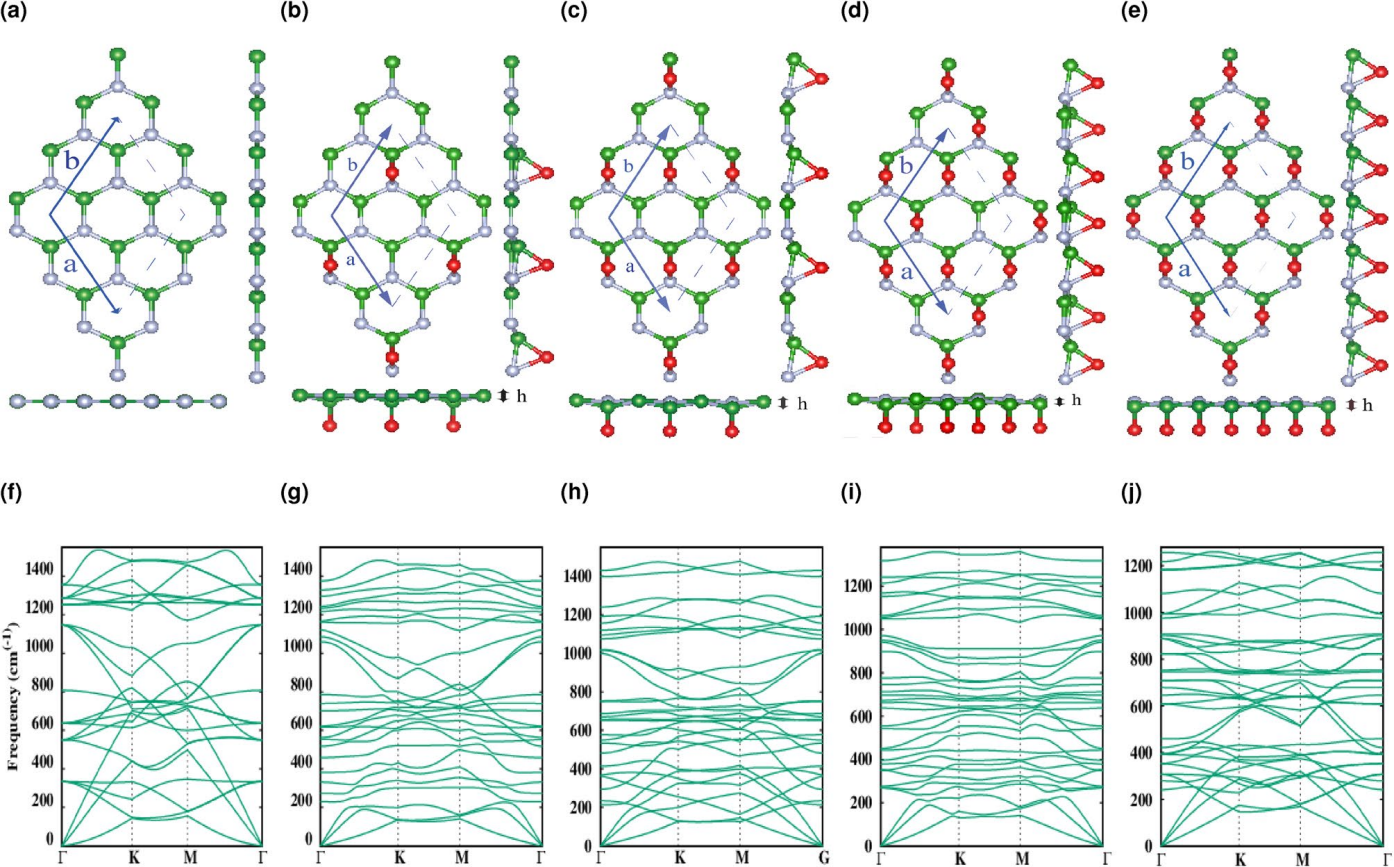
(**a**) Top and side view of pristine hBN. (**b**–**e**) Top and side view of $\Theta$ = 1/8, 2/8, 3/8, and 4/8 oxygen functionalized hBN. (**f**) Phonon dispersion for pristine hBN along different high-symmetry paths. (**g**–**j**) Phonon dispersion for $\Theta$ = 1/8, 2/8, 3/8, and 4/8 oxygen functionalized hBN along different high-symmetry paths.

We consider a ($2\times 2$) supercell for the light to dense oxygen coverage denoted as $\theta$ = 1/8, 2/8, 3/8, and 4/8 bridge-bonded oxygen atoms on the hBN surface. The fully relaxed pristine and functionalized hBN structures are displayed in Fig. 1a–e.

**Figure 2:**
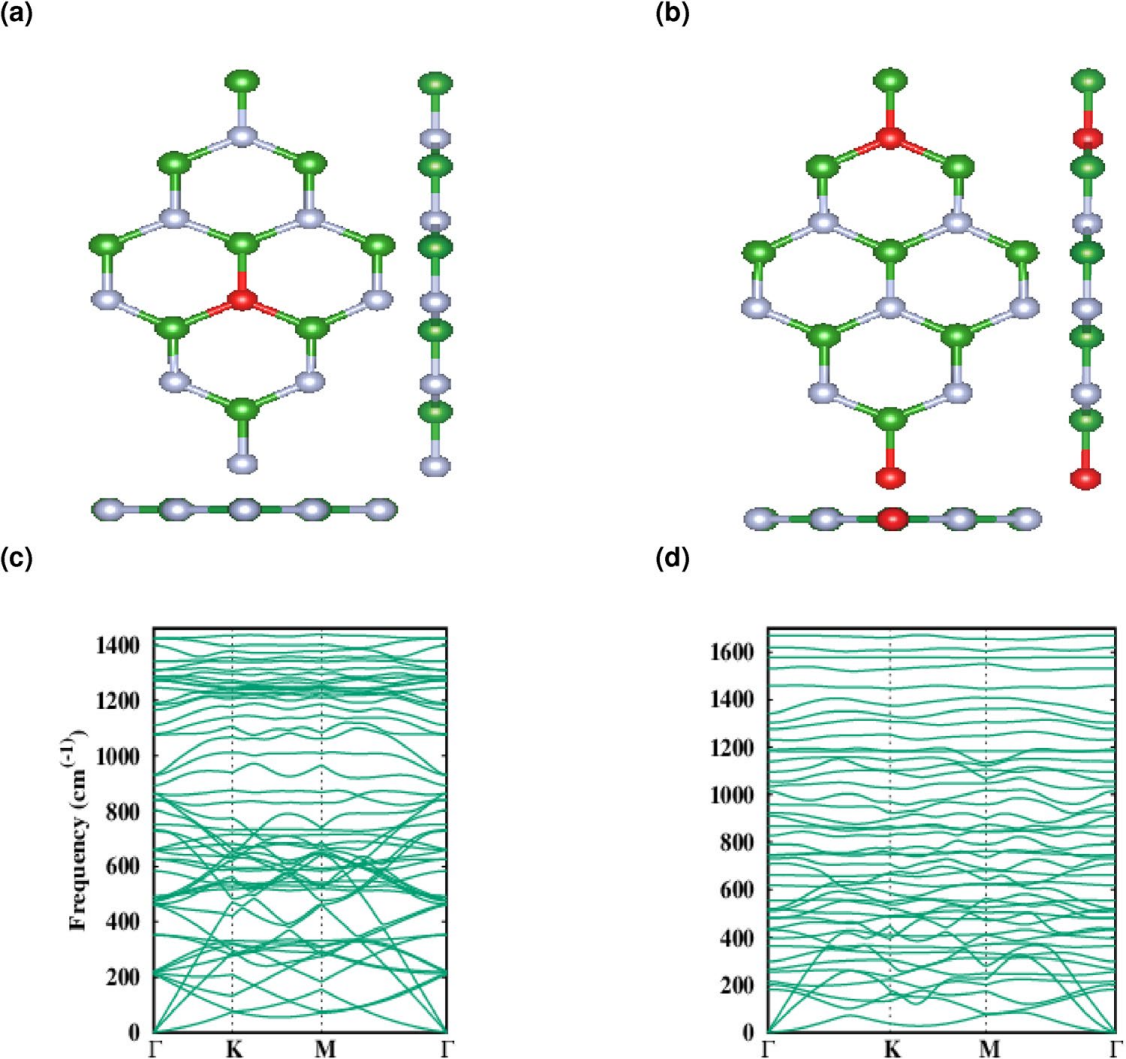
(**a**, **b**) Top and side views of 5.5 and 11% oxygen doped hBN. (**c**, **d**) Phonon dispersion for 5.5 and 11% oxygen doped hBN along different high-symmetry paths.

Next, we deal with the doping of oxygen atoms which are substituted the nitrogen atoms as it is found that by substituting the boron atoms, the structure would no longer keep its shape and collapse^29^. Here we consider low level doping concentrations denoted as $\Delta$ = 5.5 and 11% which the fully relaxed structures are shown in Fig. 2a,b, respectively. Table 2 summarizes the relaxed parameters for the doped structures which is showing a slightly change in the lattice parameters due to the comparable atomic radius of oxygen with born and nitrogen. But different band-gaps in spin up and down electronic bands.

**Table 2:** Relaxed structural parameters for $\Delta$ = 5.5 and 11% of oxygen doped hBN.

Structure	a = b (Å)	$\gamma \,(^{\circ })$	$E_g\,(up)$ (eV)	$E_g\,(down)$ (eV)
$\Delta$ = 5.5	7.549	120	4.357	4.158
$\Delta$ = 11	7.550	120	4.133	3.981

## Discussion

Figure 1a shows the optimized structure of pristine hBN. The optimized lattice vectors a = b = 5.027 Å are in good agreement with reported experimental and theoretical results^30,31^. Our considered supercell is specified by blue dashed lines and contains 4 pairs of boron and nitrogen atoms situated in the hexagonal form. Four different structures with oxygen absorption ranging from 1 to 4 per 8 atoms ($\Theta$ = 1/8, 2/8, 3/8, and 4/8) are presented in Fig. 1b–e. In which oxygen atoms are bridge bonded to boron and nitrogen atoms at the top midway. According to Table 1 [Lattice constants (a, b), the angle ($\gamma$) between a and b, and the buckling for $\theta$ = 1/8, 2/8, 3/8, and 4/8 functionalized structures.] bridge bonded oxygen atoms on one side produce 0.28, 0.29, 0.29, and 0.20 Å distortion on functionalized systems, respectively.

To examine the stability of considered structures energetically we calculated the binding energy ($E_b$) according to the equation: $E_b=E_{BN}+E_O-E_{BN+O}$ where $E_{BN}$ is the total energy of pristine hBN, $E_O$ is the total energy of the free oxygen atom which is calculated in the vacuum, and $E_{BN+O}$ is the total energy of oxygen-absorbed hBN structure. As this equation shows, the more negative the binding energy is, the more stable the structure is. The reported binding energies are indications of the stability of all configurations especially for $\Theta$ = 4/8, it is the most stable.

### Electronic properties

**Figure 3:**
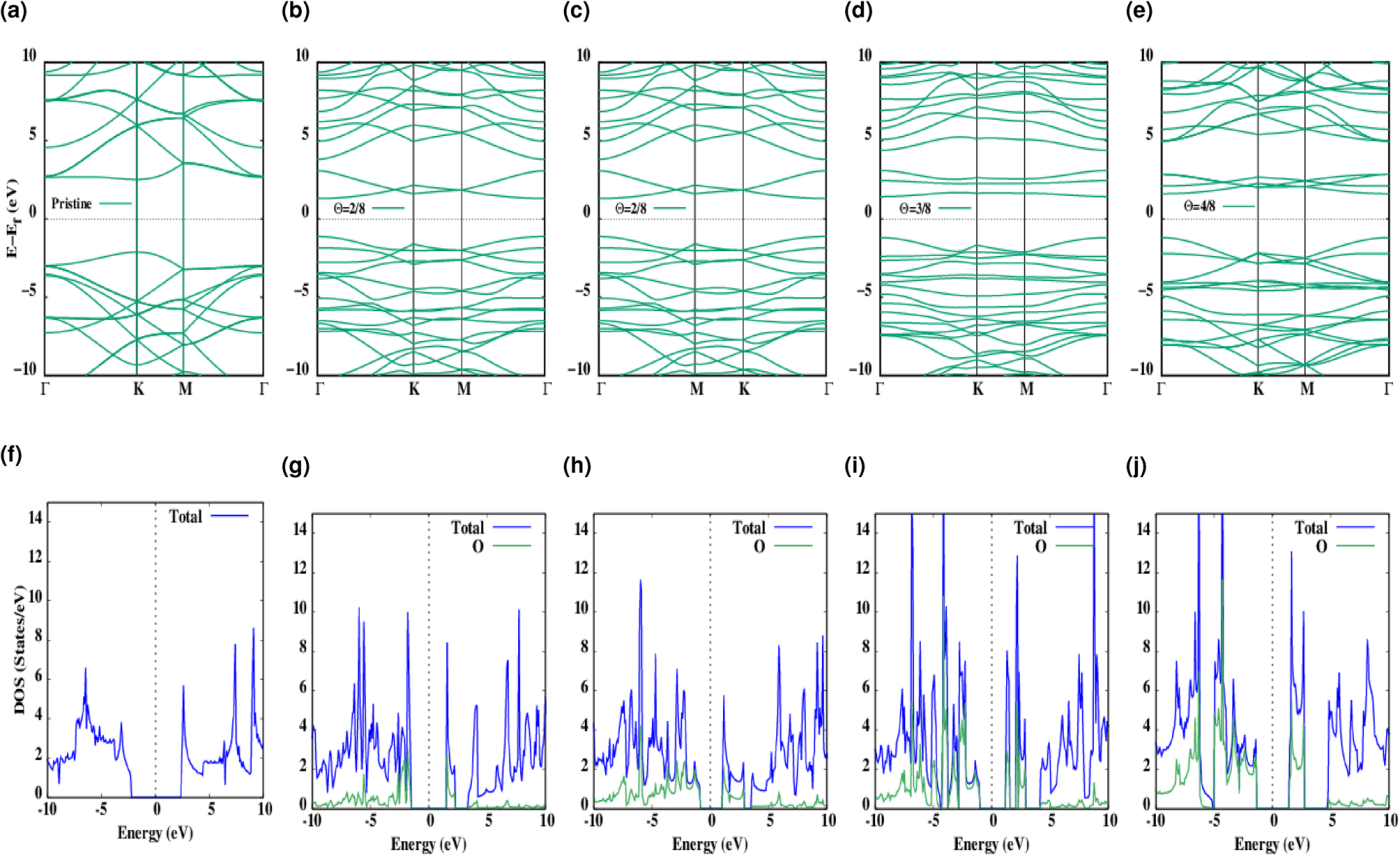
(**a**) The energy band structure of pristine hBN which is folded to the ($2 \times 2$) supercell. (**b**–**e**) The energy band structure of $\Theta$ = 1/8, 2/8, 3/8, and 4/8 oxygen functionalized hBN. (**f**) Corresponding TDOS (solid blue line) of pristine hBN. (**g**–**j**) TDOS (solid blue line) and PDOS projected to the oxygen atoms (solid green line) of $\Theta$ = 1/8, 2/8, 3/8, and 4/8 oxygen functionalized hBN. Zero of the band structure and DOS energy is set to the Fermi energy, $E_f$ which is shown by a dashed black line. The zero of energy is set to the Fermi level.

To make it clear what is the effect of functionalization on the electronic properties of such materials, the band structure of pristine hBN is depicted in Fig. 3a. As it is clear from Fig. 3b–e the absorption of adatoms with different concentrations lead to the rather flat bands and consequently can be considered as the localized impurity state. And from the band dispersion it can be seen that in the areas near the edges of the valence band maximum, oxygen is strongly bonded to the boron and nitrogen.

On the other hand, to investigate the change of electronic bonding after oxygen functionalization, we provided the corresponding total density of states (TDOS) and the partial density of states (PDOS) graphs projected on oxygen atoms in Fig. 3f–j where it is observed that the elevation of boron and nitrogen atoms after oxygen adsorption changes the $sp^2$ hybridization in hBN surface. As it is mentioned before the same behavior in oxygen and total contributions in DOS shows the strong hybridization in the out of plane orbitals.

**Figure 4:**
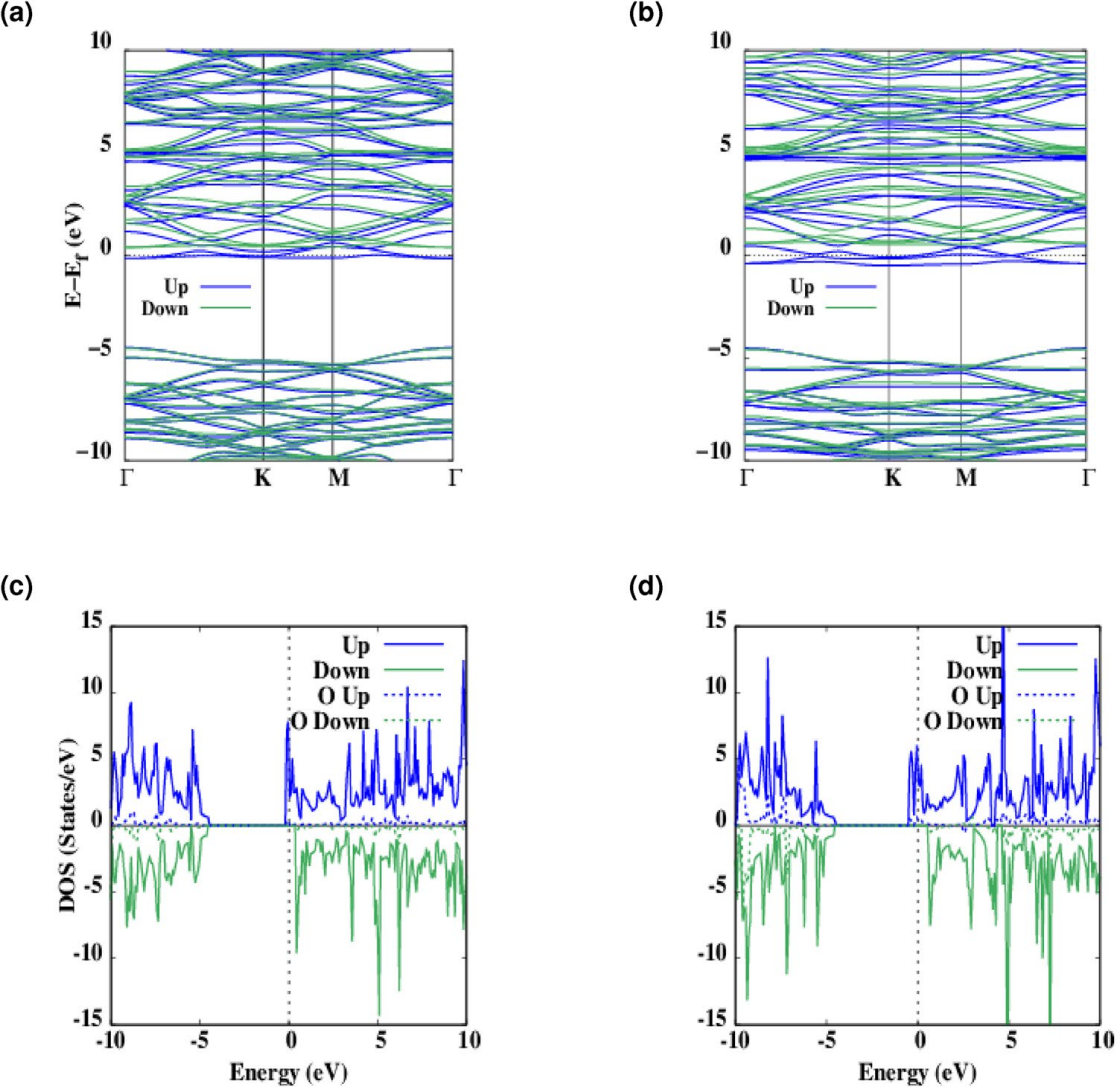
(**a**, **b**) The spin-polarized energy band structure (solid blue, green line for a spin up, down, respectively) of $\Delta$ = 5.5 and 11% oxygen doped hBN. (**c**, **d**) The corresponding spin-polarized total density of states (DOS) (blue, green line for the spin up, down, respectively) and Projected density of states (DOS) (dashed blue, green line for the spin up, down, respectively) to the oxygen atoms. Zero of the band structure and DOS energy is set to the Fermi energy, $E_f$ which is shown by the dashed black line. The zero of energy is set to the Fermi level.

Here we have considered low level doping concentrations ($\Delta$ = 5.5 and 11%) by which unpaired electrons has been introduced to the structures and the adequate degree of ferromagnetism is evident, which in turn changes the electronic properties of oxygen doped hBN and consequently the appearance of spin splitted bands. From Fig. 4a,b one can see that spin down channel exhibit a wider band-gap than spin up does. As PDOS curves in Fig. 4c,d show there are more electronic states in the spin up channels which are mainly contributed by the O 3d orbitals and by increasing the level of doping there are more occupied states.

**Figure 5:**
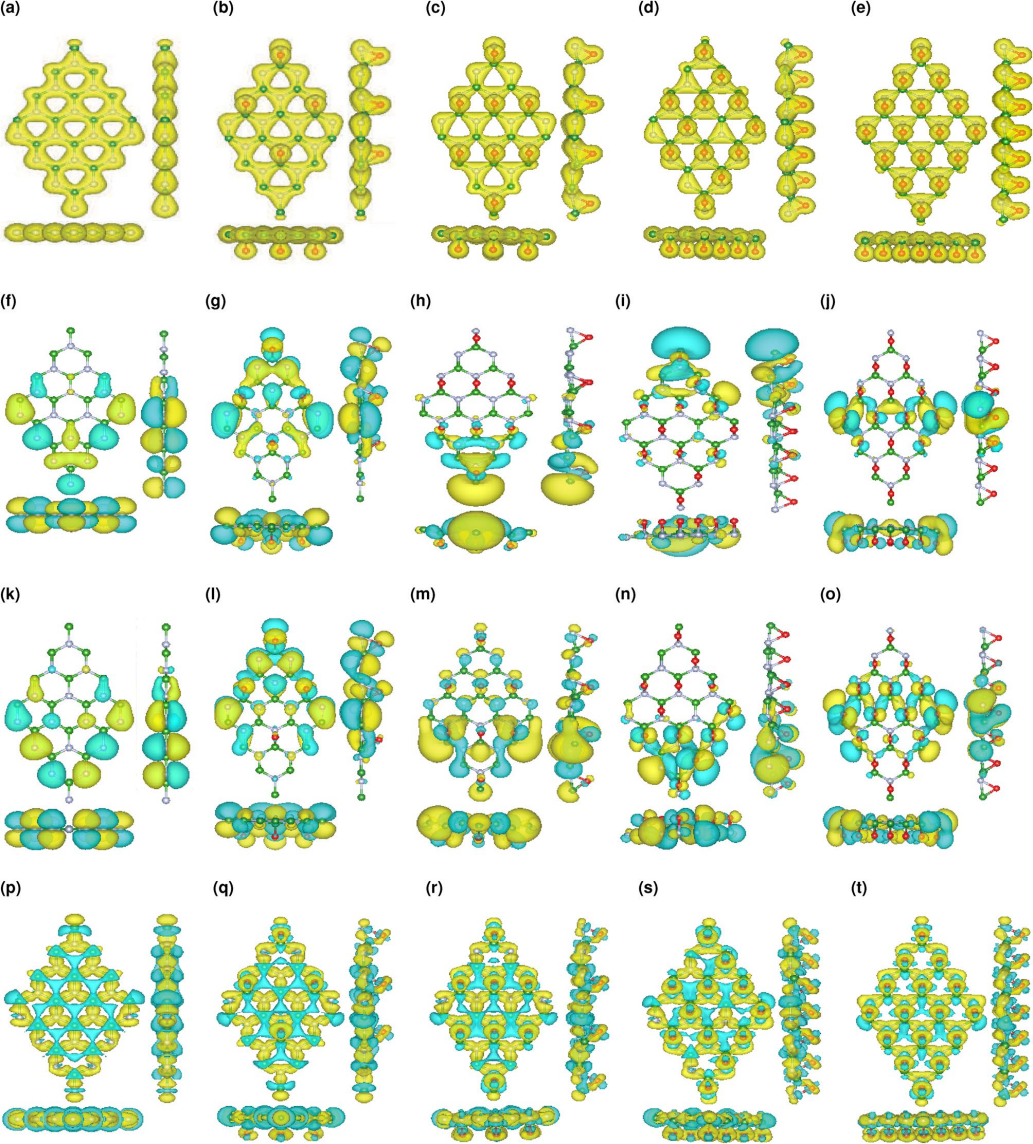
(**a**) Top and side view of total charge density for the pristine hBN. (**b**–**e**) Top and side views of total charge density for the $\Theta$ = 1/8, 2/8, 3/8, and 4/8 functionalized hBN. (**f**) Top and side view of charge density at VBM for the pristine hBN. (**g**–**j**) Top and side views of charge density at VBM for the $\Theta$ =  1/8, 2/8, 3/8, and 4/8 functionalized hBN. (**k**) Top and side view of charge density at CBM for the pristine hBN. (**l**–**o**) Top and side views of charge density at CBM for the $\Theta$ = 1/8, 2/8, 3/8, and 4/8 functionalized hBN. (**p**) Top and side of charge density difference for the pristine hBN. (**q**–**t**) Top and side views of charge density difference for the $\Theta$ = 1/8, 2/8, 3/8, and 4/8 functionalized hBN. The yellow regions indicate the positive value (electron accumulation) whereas, the cyan regions represent the negative values (electron depletion) The isosurface of charge densities is equal to 0.01 e/Å$^3$.

Figure 5a–e represent the total charge for the considered supercell of pristine hBN and $\Theta$ = 1/8, 2/8, 3/8, and 4/8 oxygen functionalized hBN, which is uniformly spread all over the surface of these nanostructures and there is no localization of charge. Figure 5f–j show The conduction band minimum (CBM) and Fig. 5k–o show the valence band maximum (VBM) charge densities for the pristine hBN and $\Theta$ = 1/8, 2/8, 3/8, and 4/8 oxygen functionalized hBN at specific k-points. These charge densities originate from the even contribution of in-plane and out-of-plane orbitals since from the Fig. 5a–e it is clear that the electron effective mass is uniformly localized around all the atoms and decreased as the overlapping of the nearest-neighbor atomic wave functions is more dominated. The theoretical calculation of the charge deformation density of the considered structure makes it possible to visualize a picture of the electron cloud expansion, especially for the expansion of electron around the adsorbed oxygen atoms, which leads to an effective shorter O-B/N length and consequently the crystal field strength and spectral shift will be increased^32^. Figure 5p–t provide a clear vision of charge density difference for the considered structures.

**Figure 6:**
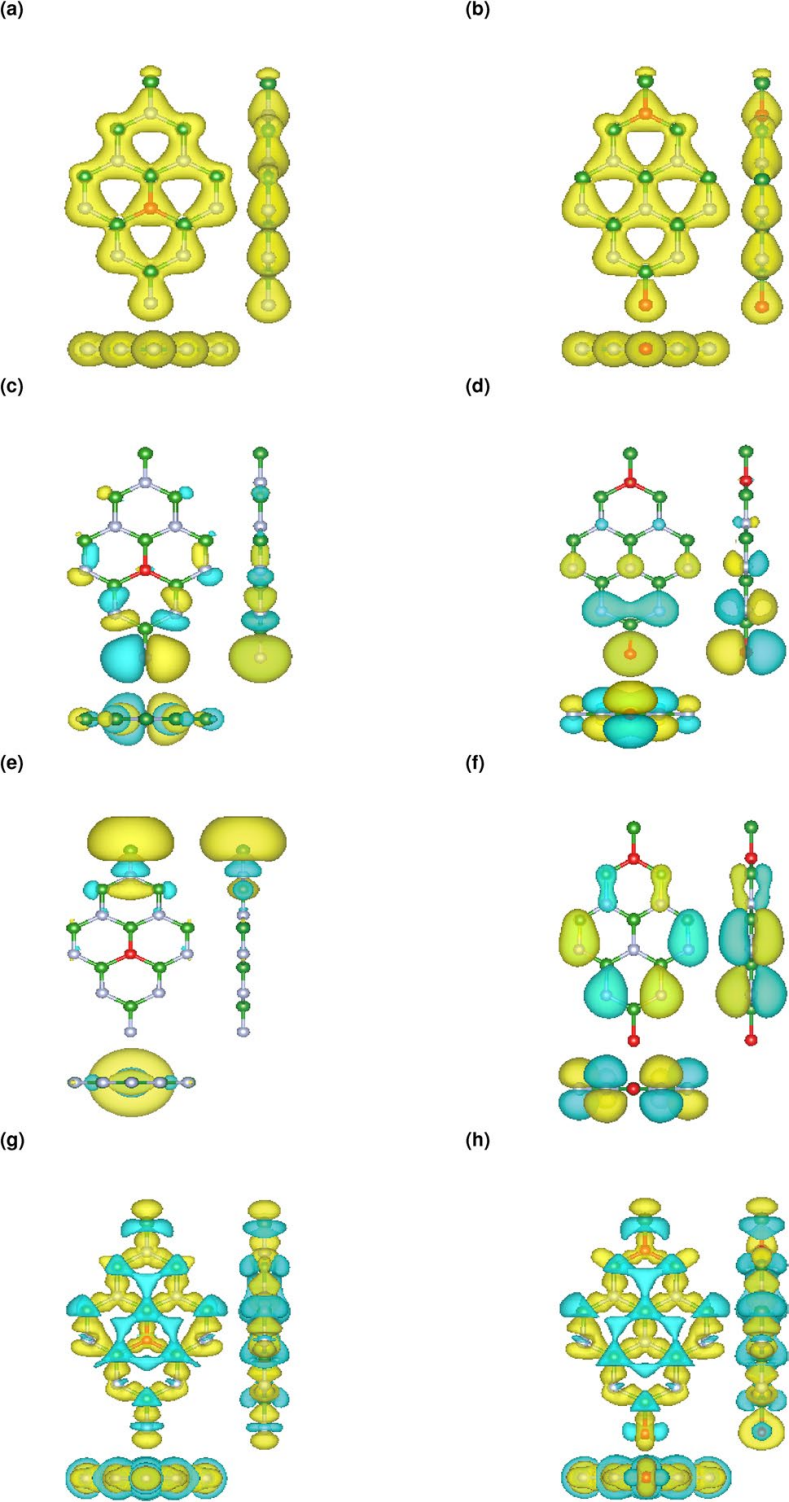
(**a**, **b**) Top and side views of total charge density for $\Delta$ = 5.5 and 11% Oxygen doped hBN. (**c**, **d**) Top and side views of charge density at VBM for $\Delta$ = 5.5 and 11% Oxygen doped hBN. (**e**, **f**) Top and side views of charge density at CBM for $\Delta$ = 5.5 and 11% Oxygen doped hBN. (**g**, **h**) Top and side views of charge density difference for $\Delta$ = 5.5 and 11% Oxygen doped hBN. The yellow regions indicate the positive value (electron accumulation) whereas, the cyan regions represent the negative values (electron depletion) The isosurface of charge densities is equal to 0.01 e/Å$^3$.

Next, we provide the total charge for the considered doped structures in Fig. 6a, b. As Asif et al.^33^ stated oxygen atoms are playing the role of electron donors to the hBN sheet. Figure 6c,d represent the CBM and Fig. 6e,f show VBM charge densities for the doped structures. As it is clear from the edges of doped configuration, there are more localized states with respect to the functionalized systems, since by substituting nitrogen atoms with oxygen atoms, unpaired electrons and consequently out of plane orbitals are introduced to the surface. Also, according to the Fig. 6g,h there is a difference in the charge deformation with respect to the functionalized cases as indication of different electron cloud expansion.

### Dynamical stability

To investigate the dynamical stability of each functionalized/doped structure, we examined them with respect to their phonon band dispersions. In all cases, a minimum of three acoustic branches are linear around the center of the Brillouin zone ($\Gamma$ point) point, which is an indication of the 2D nature of the considered materials.

The phonon dispersion of pristine/ functionalized hBN for unit cells of 8, 9, 10, 11, 12 atoms, respectively are illustrated in Fig. 1f–j, which for more clarity we provide them along with the relaxed structures. As it can be seen these illustrations not only bring about to 3N phonon branches i.e. 24, 27, 30, 33, 36, but also, they are similar to that of the unit cell, however, there are artificially generated (degenerated) phonon branches which one can attribute them to the six main branches of the original unit cell.

Our resulting phonon modes are showing no negative frequency appearing along with the high-symmetry directions of the Brillouin zone, hence the predicted structures are stable at T = 0 K. Due to the large supercell considered in our phonon calculation in order to make it possible to take into account different percentages of coverage/impurity, the band folding effect in the Brillouin zone is playing a significant role in band arrangement.

For the low doping concentrations, we have calculated the phonon dispersion without any negative frequency. Figure 2c,d along with the relaxed structure indicate that up to this level of doping, hBN is keeping its stability as it has been shown experimentally through Weng et al. work^29^.

### Thermal stability

**Figure 7:**
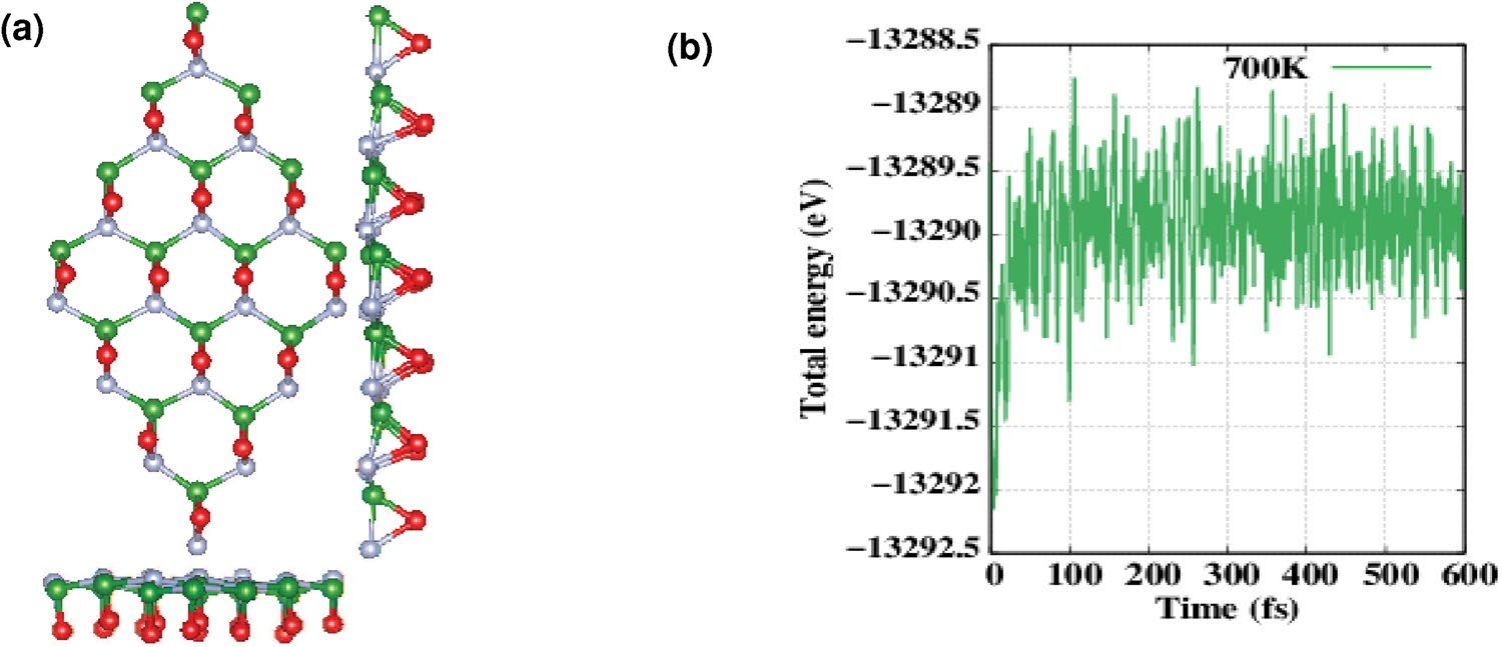
(**a**) Simulated structure of 2D functionalized hBN with $\Theta$ = 4/8 after molecular dynamics annealing at 700 K for 600 fs. (**b**) Fluctuation of total energy ($12 \times 12 \times 1$ supercell) during NVT ab initio MD simulation at 700 K.

The structural stability of a nanomaterial is an important criterion for its practical applications. The NVT (Canonical ensemble with constant number (N), volume (V), and temperature (T)) ab initio MD simulation at 700 K was done to survey the thermal stability of hypothesized structures. According to Fig. 7a,b for the high coverage structure we can see the exhibition of vigorous thermal stability at higher temperatures up to 600 fs, which is due to the strongly bonded boron and nitrogen in the hBN lattice and also high-stability of oxygen atoms bonded to hBN surface as it can be inferred from the phonon dispersion. In this regard, although there are more surface distortion, larger bond lengths, and changed bond angles when keeping the structure at high temperature, still, all bridge bonded oxygen atoms remain unchanged. Moreover, for the whole process, there is no isolated oxygen atom and they stayed bonded. We have increased the temperature further and at 1400 K this structure started to lose the bonds after about 40 fs so we did not continue the calculations.

### Optical properties

**Figure 8:**
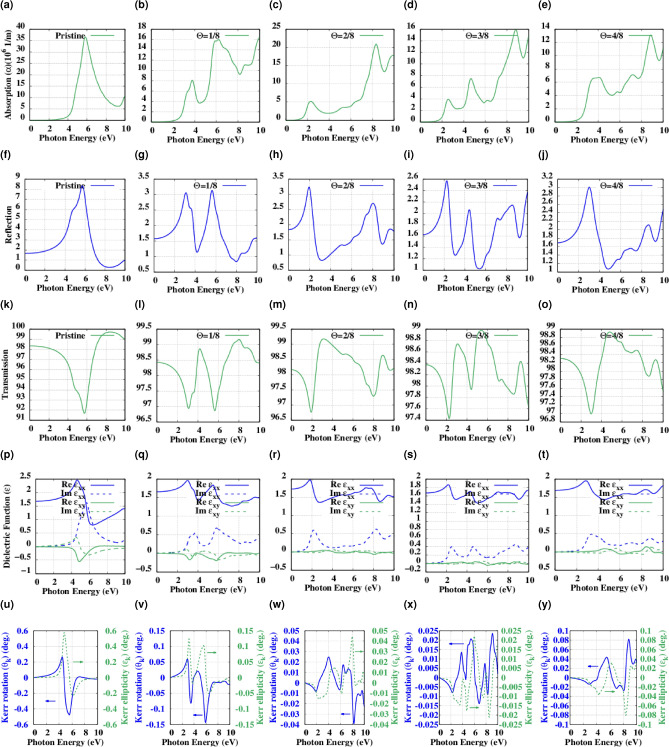
(**a**) The optical absorption spectrum for the pristine hBN. (**b**–**e**) The optical absorption spectrum for the $\Theta$ = 1/8, 2/8, 3/8, and 4/8 functionalized hBN. (**f**) Calculated spectra of reflection for Pristine hBN. (**g**–**j**) Calculated spectra of reflection for the $\Theta$ = 1/8, 2/8, 3/8, and 4/8 functionalized hBN. (**k**) Calculated spectra of Transmission for Pristine hBN. (**l**–**o**) Calculated spectra of transmission for the $\Theta$ = 1/8, 2/8, 3/8 and 4/8 functionalized hBN. (**p**) Calculated real part (solid lines) and imaginary part (dashed lines) of both diagonal $\varepsilon _{xx}$ (blue) and off-diagonal $\varepsilon _{xy}$ (green) dielectric functions of the pristine hBN. (**q**–**t**) to calculated real part (solid lines) and imaginary part (dashed lines) of both diagonal $\varepsilon _{xx}$ (blue) and off-diagonal $\varepsilon _{xy}$ (green) dielectric functions of the $\Theta$ = 1/8, 2/8, 3/8, and 4/8 functionalized hBN. (**u**) Kerr angle $\theta _K$ (left, blue solid) and Kerr ellipticity $\chi _K$ (right, green dashed) of the pristine hBN. (**v**–**y**) Kerr angle $\theta _K$ (left, blue solid) and Kerr ellipticity $\chi _K$ (right, green dashed) of the $\Theta$ = 1/8, 2/8, 3/8, and 4/8 functionalized hBN in the P-MOKE configuration.

In Fig. 8a–e we compare the optical absorption spectrum which is computed for pristine hBN and its functionalized structures. The pristine hBN shows the first absorption peak at 5.82 eV (213 nm). For functionalized structures the first absorption peak is located at 3.78 eV (328 nm), 2.30 eV (539), 2.58 eV (480 nm) and 4.09 eV (303 nm) which are demonstrating the smooth continuous process of redshift proportion with increasing oxygen concentration (see Table 3) on the surface and this is in correlation with the smaller band-gap^34^. On the other hand, by increasing the oxygen percentage on the surface the absorption peaks are decreasing in intensity which is an indication of optical transition and redistribution of hBN’s electron-hole density near the excited states^35^.

**Table 3:** First optical absorption peak (OA), maximum values of transmission and reflection, dielectric function peak (DF), and maximum value of Kerr rotation for the pristine hBN, different concentration of surface oxygen functionalization, and different levels of doping.

Structure	OA (eV)	Reflection ($\%$)	Transmission ($\%$)	(DF) (eV)	Kerr rotation ($^{\circ }$)
Pristine	5.82	8.32	99.71	5.66	0.47
$\theta$ = 1/8	3.78	3.13	99.16	3.72	0.14
$\theta$ = 2/8	2.30	3.23	99.17	2.16	0.03
$\theta$ = 3/8	2.58	2.57	98.97	2.50	0.02
$\theta$ = 4/8	4.09	3.00	98.93	3.37	0.08
$\Delta$ = 5.5	0.48	22.71	77.28	0.19	0.12
$\Delta$ = 11	0.64	37.29	62.70	0.21	0.15

Figure 8f presents the calculated reflection spectra of pristine hBN which resonates around 5.72 eV (216 nm) in possession of an almost symmetrical line shape. Figure 8g–j depict the calculated reflection spectra for the $\Theta$ = 1/8, 2/8, 3/8, and 4/8 functionalized hBN, which resonate around 3.09, 1.94, 2.27, and 3 eV, respectively for the first time. Also, the transmission spectra of pristine hBN and the $\Theta$ = 1/8, 2/8, 3/8, and 4/8 functionalized hBN was characterized and it shows the maximum value of 99.71% for the pristine hBN and 99.16, 99.17, 98.97, and 98.93 which shows that the transparent nature of these materials is kept untouched, Fig. 8k–o. All of our calculated values are summarized in Table 3 for a better insight.

**Figure 9:**
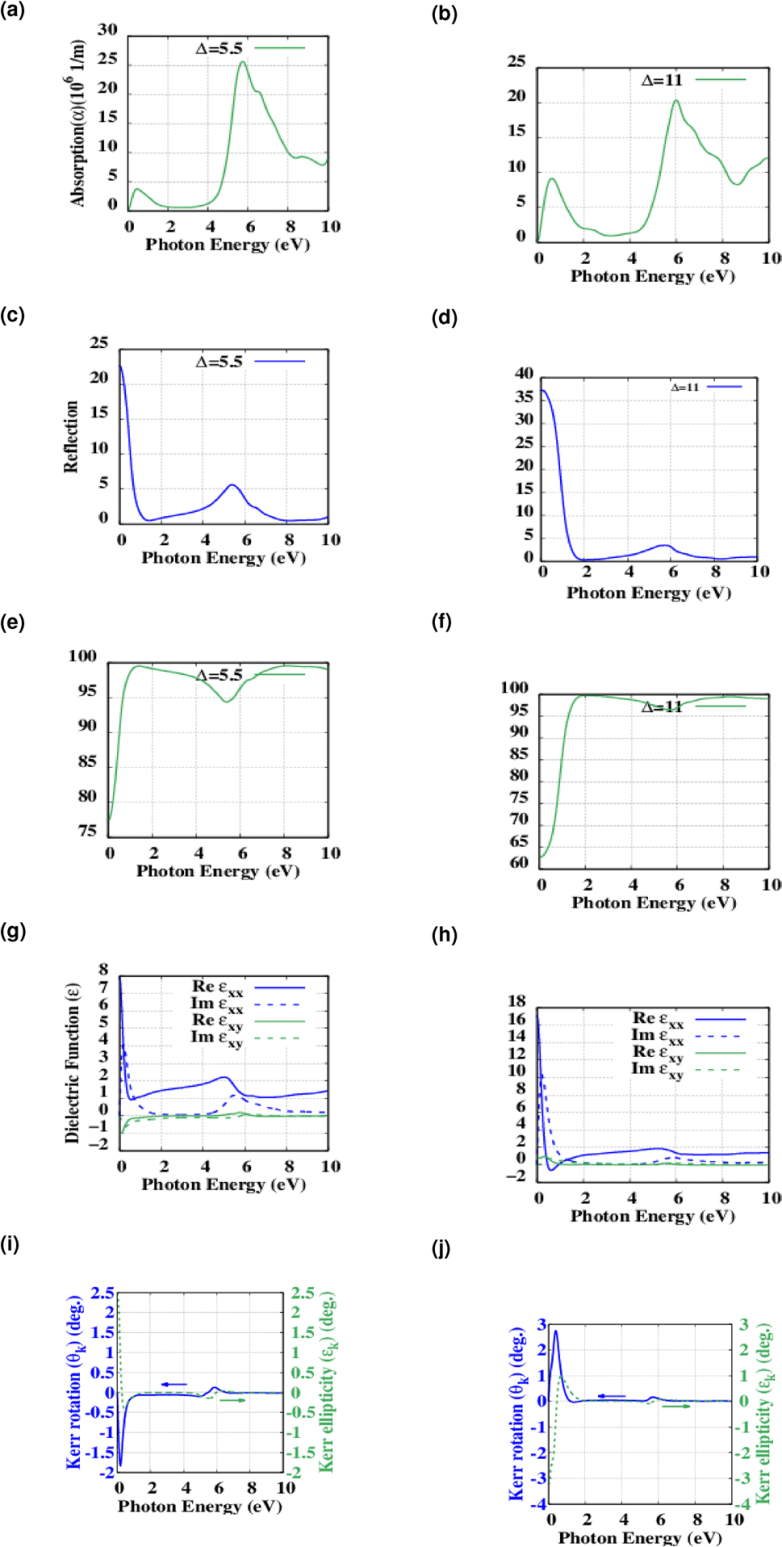
(**a**, **b**) The optical absorption spectrum of $\Delta$ = 5.5 and 11% Oxygen doped hBN. (**c**, **d**) Calculated spectra of reflection for $\Delta$ = 5.5 and 11% Oxygen doped hBN. (**e**, **f**) Calculated spectra of reflection for $\Delta$ = 5.5 and 11% Oxygen doped hBN. (**g**, **h**) calculated real part (solid lines) and imaginary part (dashed lines) of both diagonal $\varepsilon _{xx}$ (blue) and off-diagonal $\varepsilon _{xy}$ (green) dielectric functions of $\Delta$ = 5.5 and 11% Oxygen doped hBN. (**i**, **j**) Kerr angle $\theta _K$ (left, blue solid) and Kerr ellipsity $\chi _K$ (right, green dashed) of $\Delta$ = 5.5 and 11% Oxygen doped hBN in the P-MOKE configuration.

The diagonal and off-diagonal elements of dielectric function for the functionalized structures are presented in Fig. 8p–t. For pristine hBN the imaginary part of the dielectric function exhibits a sharp peak at 5.66 eV and for the functionalized structures this peak is shifted toward lower energies. As for surface functionalization, we adopted optical properties for the different levels of oxygen doping. Figure 9a,b showing modified optical absorption in which the first absorption peak is further moved to the UV region by exciting near 0.48 and 0.64 eV for $\Delta$ = 5.5 and 11, respectively. which clearly depicts the effect of oxygen doping on the optical band-gap and also the threshold of optical absorption. Also from the Fig. 9c,d,e,f and also Table 3 one can infer that the reflection capability is increased therefore these structures have lost their transparency to some degree which are due to the lower level of recombination in the valence band and leads to the losing transparency in these material’s to an order of magnitude. Moreover, The diagonal and off-diagonal elements of dielectric function for the doped structures are presented in Fig. 9g,h. The imaginary part of the dielectric function exhibits a sharp dip near 0.19 eV associated with BNO TO phonons and the absorption peak near 0.48 eV is related to the LO phonons^36^. In the case of $\Delta$ = 11% of oxygen doping, as it can be seen from the real part of the diagonal element of the dielectric function, there is a dip in the intensity that attains a comparatively good negative value of $-0.62$ which is a good indication of plasmonic excitation near 0.61 eV^37^.

### Magneto-optical Kerr effect

As Ataka et al.^30^ concluded, oxygen functionalization is introducing a FM phase to 2D hBN which is indeed a manifest of spin-interfering property of such manipulated materials. Mainly, theoretical accounting for MO effects manifests in investigating the diagonal and off-diagonal energy/frequency-dependent macroscopic dielectric functions^28^. In the process of reflecting a linearly polarized light from a magnetized material, MOKE appears to be observed in terms of the Kerr angle, $\theta _K$. In addition, Kerr ellipticity is defined by an angle, $\eta _K$ which shows the level of polarization directly proportional to the magnetization component in the elliptically polarized light^1,24^. Among three different orientations of magnetization vector $\varvec{M}$ of the considered material, polar type is more technically popular. Where the incident light and the direction of the magnetization vector are both perpendicular to the plane of the magnetized material. For this symmetry, the Kerr effect is given by^24^

1 $$\begin{aligned} \theta _K(\omega )+i\eta _K(\omega )=-\dfrac{\sigma _{xy}(\omega )}{\sigma _{xx}(\omega )\sqrt{1+(4\pi /\omega )\sigma _{xx}(\omega )}} \end{aligned}$$

where $\sigma _{xx}$ and $\sigma _{xy}$ stand for the diagonal and off-diagonal components of the complex optical conductivity, respectively. In this relation, the sign convention has been considered so that for clockwise rotation of the incoming beam, $\theta _K$ is positive. The relation between $\sigma _{xx}$ and $\sigma _{xy}$ with the dielectric tensor, $\varepsilon _{\alpha \beta }$ is as follows^24^

2 $$\begin{aligned} \varepsilon _{\alpha \beta }(\omega )=\delta _{\alpha \beta }+\dfrac{4\pi i}{\omega }\sigma _{\alpha \beta (\omega )} \end{aligned}$$

after some simplification and considering our polar specific case the final relation for the Kerr effect will be^27^

3 $$\begin{aligned} \theta _K(\omega )+i\eta _K(\omega )=\dfrac{i\varepsilon _{xy}}{\sqrt{\varepsilon _{xx}(1-\varepsilon _{xx})}} \end{aligned}$$

where $\varepsilon _{xx}$ and $\varepsilon _{xy}$ stand for the diagonal and off-diagonal elements of dielectric tensor, respectively. In order to evaluate the Kerr effect, two important related spectral quantities, optical conductivity tensor, and dielectric tensor come into consideration. As it can be seen from the equation and provided Figures, the Kerr angle $\theta _K$ (Fig. 8u–y, solid blue curve) has the same trend as the Imaginary part of $\varepsilon _{xy}$ (Fig. 8p–t, dashed green curve) and therefore affects by the optical excitations; besides, the Kerr ellipticity $\chi _K$ (Fig. 8u–y, dashed green curve), is related to the Real part of $\varepsilon _{xy}$ (Fig. 8p–t, solid green curve). In our simulations for the pristine hBN, we achieved $-0.47^{\circ }$ (8.20 mrad) at photon energy 5.58 eV for the Kerr rotation denoted as $\theta _K$, which is a good performance dealing with a nonmagnetic material. Moreover, we expect a sign change of $\theta _k$ near 4.48 eV. As we increase the oxygen atom absorbed on the surface, the number of excitation frequencies of photons is accordingly increased. So that for $\Theta$ = 1/8, 2/8, 3/8, and 4/8 oxygen functionalized hBN, we have a wide spectrum of MO resonance ranging from $\theta _K=-0.14^{\circ }$ (2.44 mrad) to $\theta _K=-0.01^{\circ }$ (0.17 mrad) for $\Theta =1/8$ and $\Theta =4/8$, respectively. However, for the lower photon energies, we have several sign changes of $\theta _k$ with lower level of magnitudes which are rather compatible with the oxygen concentration. The maximum values of Kerr rotation for each structure are provided in Table 1.

On the other hand, as predicted before different levels of doping increases the Kerr rotation up to $\theta _K=0.12^{\circ }$ and $\theta _K=0.15^{\circ }$ for $\Delta =5.5$ and 11% (Fig. 9i,j), respectively as another indication of the ferromagnetic nature of underlying materials which have been proved experimentally through electron paramagnetic resonance (EPR) scanning^29^. As Weng et al. stated this attributes to the substitution of oxygen atoms with Nitrogen resulting in unpaired electrons.

## Conclusions

We have performed electronic, optical, and magneto-optical calculations for the proposed hBN based surface engineered nanomaterials using DFT method. It has been shown that by different values of surface functionalization, the band-gap decreases from 4.67 eV to a desired value of 2.43 eV depending on the arrangement of adsorbed atoms and the different types of orbital couplings. Subsequently, we investigated the thermo/mechanical stability of such materials leading to the confirmation of dynamical stability of 6 proposed functionalized/doped structures. Besides, keeping high oxygen coverage structure up to 600 fs in the temperature of $700^{\circ }$ without changing the bonding nature, which can be extended to a longer period of time shows the high level of thermal stability of these materials.

As compared to Pristine hBN, the absorption peak position for $\Theta$ = 2/8 shows red shift of 3.52 eV at maximum level. For the first time, we have calculated Kerr rotation for surface engineered and extrinsic ferromagnetic material up to $\theta _K=0.15^{\circ }$ which is comparable with Heusler alloys^24,38^. As Catarina et al. stated^39^ the Kerr rotation is the sum of different curves of spin and valley components which have resonant peaks around the absorption spectra so it is highly related to the effective band-gap of the materials. Also, we showed that MO signals in hBN and its functionalized/doped nanostructures are strong which can be extensively used as an optical switch. Interestingly, we have found that for the 11% doped structure a small value of surface plasmon is created near 0.61 eV which is a valuable improvement in a 2D material that can lead to wider applications.

Although, oxygen functionalization caused the hBN to lose the maximum transparency by about 1%, it still poses an accepted level of transmission (up to 98.93 for the high oxygen coverage) which makes it a proper material for microelectronic and opto-electronic applications. Furthermore, as there are several reports^40,41^ on the induced magnetic properties for surface modification by fluorine and carbon, our future aim is to consider magneto-optical investigation considering such elements.

## Methods

We performed first-principles, spin-polarized calculations on the hBN-derived materials. The geometry optimizations and electronic structure calculations were performed under certain conditions where the total energy difference lowered down to $10^{-5}$ eV and component forces less than $10^{-3}$ eV/Å act on each atom using the SIESTA DFT-based code^42,43^. We have used a double-$\zeta$ polarized basis set for all individual atoms and the generalized gradient approximation (GGA) in the scheme of Perdew, Burke, and Ernzerhof (PBE)^44^ was conducted for the exchange-correlation functional. We considered different concentrations of adatoms where the electronic, optical, and magnetic properties of hBN structures developed accordingly.

In our calculations, The Brillouin zone integrations are performed on the Monkhorst-Pack^45^ *k*-point grid of $12\times 12 \times 1$, for the electronic structure calculations. The real-space Fourier expansion of the electron density is cut at 470 Ry. Also, charge-transfer analysis was performed using the Denchar post-processing tool. For calculating MOKE we have performed some programming to include our resulting data.

Optical properties Calculations were performed using the plain-wave pseudopotentials as implemented in the OpenMx 3.9 package^46,47^. This code uses norm-conserving pseudopotentials to evaluate eigenfunctions and eigenvalues of Kohn-sham equations, and also (PBE-GGA) is applied to consider the exchange-correlation functional. The converged energy cutoff and Monkhorst-Pack kpoint sampling was chosen to be 560 Ry and $18\times 18\times 1$, respectively.

As the conductivity and dielectric function can be calculated based on the Kubo-Greenwood formula in this work we did not take into account the electron-hole interaction which is causing excitonic effects^48^. We considered a sufficiently large vacuum pad of 20 Å for these periodic structures to avoid any interaction between adjacent layers and the atomic positions were optimized using a quasi-Newton algorithm, where the force acting on each atom was less than $10^{-3}$ eV/Å. For this purpose, we placed oxygen atoms at an energetically minimum position in bridge cites and let all atoms in the supercell be fully relaxed in all directions.

## Data Availability

The data that support the findings of this study are available from the corresponding author upon reasonable request.
